# Rheological and Textural Properties of Apple Pectin-Based Composite Formula with Xanthan Gum Modification for Preparation of Thickened Matrices with Dysphagia-Friendly Potential

**DOI:** 10.3390/polym13060873

**Published:** 2021-03-12

**Authors:** Huaiwen Yang, Chai-Chun Tsai, Jung-Shiun Jiang, Chi-Chung Hua

**Affiliations:** 1 Department of Food Science, National Chiayi University, Chiayi 60004, Taiwan; s986249@gmail.com; 2 Department of Chemical Engineering, National Chung Cheng University, Chiayi 62102, Taiwan; kawasaky329@hotmail.com

**Keywords:** apple pectin, xanthan gum, rheological properties, texture profile, dysphagia

## Abstract

Modifying the consistency of a given edible fluid matrix by incorporating food thickeners is a common nursing remedy for individuals with dysphagia when adequate water consumption is a concern. As apple pectin (AP) offers nutraceutical benefits, properly formulated apple pectin (AP)-based thickeners featuring xanthan gum (XG) can be superior candidates for preparation of dysphagia-friendly matrices (DFMs). Our recruited DFMs exhibit fluid-like behavior (loss modulus > storage modulus, G” > G’) at lower AP concentrations (2 and 5%, *w*/*w*); they turn into weak/critical gels (G’ ≈ G”) as the concentration becomes higher (9%). In contrast, XG-DFMs display gel-like attributes with G’ > G”, even at rather low concentrations (<1%) and become more resistant to sugar, Na^+^, and Ca^2+^ modifications. The composite matrix of AP1.8XG0.2 (constraint at 2%) exhibits a confined viscosity of 278 ± 11.7 mPa∙s, which is considered a DFM, in comparison to only AP- or XG-thickened ones. The hardness measurements of XG0.6 and AP1.2XG0.8 are 288.33 ± 7.506 and 302.00 ± 9.849 N/m^2^, respectively, which potentially represent a promising formulation base for future applications with DFMs; these textural values are not significantly different from a commercially available product (*p* > 0.05) for dysphagia nursing administrations.

## 1. Introduction

The normal swallowing process in adults is divided into four phases: oral preparatory, oral transit, pharyngeal, and esophageal [1]. Oropharyngeal dysphagia, defined as difficulty swallowing, is a neurological disorder often diagnosed among the elderly, especially in patients with stroke, motor neuron disease, and Parkinson’s disease [2]. Conservative estimates suggest that 8% of the world population experiences difficulty consuming regular thin liquids because of dysphagia; regular thin liquid intake for elderly patients with dysphagia is problematic, as its turbulent and fast propagation is difficult to control during passage through the pharynx [3]. Therefore, dysphagic patients tend to develop a reluctance toward consuming the recommended amount of water/moisture from thin liquids to avoid aspiration (leading to frequent bulking or even pneumonia), exposing themselves to severe dehydration and malnutrition [4].

Edible thickeners are commonly employed to modify the consistency of food liquids for patients with dysphagia, as they make smoother and safer swallowing progression possible, as compared to thin liquids that may cause choking [5]. The viscosity of thickened food liquid must be carefully assessed to ensure safer swallowing for elderly dysphagic individuals with the expectation of maintaining their quality of life [6]. Besides, the stability of dysphagia-friendly food liquids is another important concern. Modified starches, for example, are one of the most common food thickeners. Modified-starch-oriented meals or meal replacements are usually prepared prior to serving or on a daily basis and stored at low temperatures in residential care establishments, like day hospitals and rehabilitation centers. Several issues related to the utilization of this conventional food thickener have been reported. First, modified starch for commercial formulation could undergo sedimentation with prolonged storage [7,8]. Moreover, they are susceptible to thermal degradation that results in considerable viscosity reduction, especially upon heating in a retort grade during sterilization by pressured/supersaturated steam. Lastly, before a piece of food bolus reaches the stomach through pharyngeal and esophageal phases, it is hydrolyzed by amylase in the saliva during oral preparatory and oral transit phases; consequently, the hydrolyzed residue of carbohydrates could increase the risk of inhalation cough during swallowing [6]. For these reasons, non-starch hydrocolloid gels have gained considerable interest recently as edible thickeners because of their superior stability than modified starches.

Pectin is a heterogeneous polysaccharide found in the cell wall compartment of terrestrial plants [9,10]. Partially methyl-esterified or acetylated galacturonic acids assemble to form the backbone polymers of pectin and rhamnopyranosyl residues, with neutral sugar side chains attached to the backbone [10]. Individual pectin molecules can aggregate to form three-dimensional structures and act as thickening agents in liquid food matrices [11]. Viscosity and other rheological characteristics of a thickener have long served as useful and reliable indicators for the evaluation of its modification competence for liquid food consistency as well as for the stability of the resulting hydrocolloids against physical alterations, like those encountered during the human swallowing process, when used at appropriate concentrations [12]. In this regard, the rheological properties of pectin thickened food matrices have been known to strongly correlate with its concentration and the interactions with common food ingredients like sugar and cations [12,13,14]. Pectin can be found in almost all plants, but commercially, most pectin merchandises are produced from citrus fruits and apples [15]. Apple pomace, 95% made of skin and pulp with attention drawing bioactive polyphenols exerting antioxidant activity, is left in the magnitude of several million tons, with an increase trend, annually, required for further sustainable processing or otherwise discarded as waste [16]. Once extracted from apple pomace, commercialized apple pectin is wildly used as an edible thickener or emulsifier [17]. 

Hydrocolloid gelling gums are also frequently employed as thickeners for the formulation of dysphagia-friendly matrices (DFMs) to avoid hydrolysis by the salivary amylase. Among the hydrocolloid gelling gums, xanthan gum (XG) is of great interest due to its favorable rheological characteristics and stability [18,19]. XG is a high-molecular-weight exopolysaccharide with a linear 1,4-D-glucose backbone and charged trisaccharide side chains at alternate residues [19]. The electrostatic repulsions between the charged side chains allow the disordered backbone to become highly extended. Thus, XG is often capable of modifying the rheological behavior of other hydrocolloids, given the complex structures that could be formed [18,19]. These combined features make XG an ideal candidate to replace pectin for dysphagia-friendly formulation, as Funami [18] reported that it is one of the most popular thickeners used in DFMs, second only to modified starches. Furthermore, pectin materials are considered to possess nutraceutical benefits for patients with diabetes and high cholesterol [20]. Recently, commercialized pectin products in powdered forms are commonly available as nutrition supplements, which can be fully miscible food fluids prior to serving. 

Rheological properties of commercial edible thickening formula with gum-based composite used for the nursing administration of dysphagia have been reported; of all those, the selected commercial sample in the study contained the same major ingredient, namely dextrin, [21,22]. Dextrin is basically safe and capable of promoting the consistency of edible fluid suitable for serving individuals with dysphasia; however, a rare related dietetic function with regards to dextrin has been reported, other than its contribution to the texture or structure of the fluid matrix. Some later studies on thickeners have evoked interest in starch-based formula due to the desperate need for calorie supplementation in the difficulty swallowing hurdle to normal consumption of solid bolus [23,24]. The rheological modification effect of XG on dextrin- and starch-based DFMs have been elaborately reported with promising outcomes to confine dysphasia diet standards [22,23,24,25]. Pectin is profoundly recognized as a gelling, thickening, and stabilizing agent, which also has great formulation potential to develop DFMs [26]. To the best of our knowledge, DFMs formulated by pectin-based composites have not been reported. Pectin-based DFMs could simultaneously supplement other nutraceutical benefits, therefore being deductively superior to dextrin- and starch-based DFMs beyond textural/theological confinements. As an excellent source of dietary fiber, pectin is well classified as an emerging prebiotic for international fermentation [27,28,29]. There have been additional studies indicating its positive effects in reduction of blood glucose [30,31], total cholesterol, and low-density lipoprotein [32]. Commercialized pectin products are also prefered for certain conscious ethnic groups, as they are more likely to be naturally obtained [16], comparing to chemically modified starch. Nevertheless, prior to the confined usage of a pectin-based composite to prepare DFMs, a systematic study is necessary, one that studies the rhetorical and textural properties of the general modifier (XG) as well as pectin-related basic food ingredients, such as sugar (sucrose), salt (sodium), and calcium.

As per the above justified concerns, this study aims to conduct a systematic study on the rheological and textural properties of an apple pectin (AP)-based composite formula with dysphagia-friendly potential by incorporating XG as a modifier as well as commonly available food ingredients, including sugar or sucrose, table salt or monovalent sodium ion, and divalent calcium ion, when formulated for DFMs. The results shed light on promising alternatives of existing formulations for future applications to DFMs.

## 2. Materials and Methods

### 2.1. Materials and Reagents 

The AP as powdered form (Solgar, Inc., Leonia, NJ, USA) and XG of molecule weight circa 2000 kDA (Gemfont Co., Taipei, Taiwan) were used as thickeners in this study. A commercial powdered thickener, Neo-high Toromeal III^®^ (TRM, Food-care, Inc., Tokyo, Japan) was also utilized as a control sample (1.5%) for texture profile analysis. White granulated sugar (Taisugar Co., Kaohsiung, Taiwan) and iodized table salt (Taisalt Co., Tainan, Taiwan) were purchased from local producers, and chemical-grade sucrose and sodium chloride (Sigma-Aldrich Co. Oakville, Ontario, Canada) were utilized for comparative purposes. Calcium chloride dihydrate was used as a divalent cation source (Ca^2+^) (Sigma-Aldrich Co.). Unless stated otherwise, all other chemicals in this study were of chemical grade.

### 2.2. Preparation of Model Food Matrices (MFDs)

Basic formulation ingredients (BFIs; sugar, sucrose and cations) were added to reverse osmosis water and mixed thoroughly at ambient temperature using a magnetic stirrer (Mini-P, Yotec Instruments Co., Taipei, Taiwan) at moderate speed for 3 h. The thickener bases (XG, AP, and AP-XG blend) were then dispersed in reverse osmosis water, with or without sucrose and cations. The hydrocolloids so prepared were transferred to 15 mL centrifugation tubes and stored at 4 °C for 24 h prior to the rheological measurements. Table 1 summarises the formulations of the model food matrices in this study.

### 2.3. Degree of Esterification (DE) 

The DE of AP was estimated in a way described previously [33] with only minor modifications. Briefly, 1.0 g of AP powder was thoroughly dissolved in 50 mL deionized water by stirring at ambient temperature for 2 h. Upon complete mixing, 6 drops of phenolphthalein solution (C_20_H_14_O_4_; Sigma-Aldrich Co., MO, USA) was added as an indicator and titrated with 0.1 M NaOH until the mixture turned pink, lasting for 30 s; the depletion volume of NaOH (mL) was recorded as *V*_1_. Subsequently, 10 mL of 0.25 M HCl was thoroughly mixed with the pink solution to neutralize the solution until it turned transparent, followed by a second titration of 0.1 M NaOH until the neutralized solution turned pink again for another 30 s. The second depletion volume of NaOH was recorded as *V*_2_. The DE was calculated using the following relation (Equation (1)):

(1) $$\mathrm{DE}\left(\%\right)=100\times \frac{V_{2}}{V_{1}+V_{2}}\;$$

### 2.4. Rheological Measurements and Flow Behavior Characterization 

A controlled-stress rheometer (DHR-2, TA Instruments, New Castle, DE, USA) equipped with a cone-and-plate fixture (diameter = 60 mm, cone angle = 1°, and gap size = 55 mm) and Peltier temperature controller was used at 25.0 ± 1 °C. Solvent traps were used through the entire measurement to avoid solvent evaporation. Rheological characterization was performed by the following sequences: (dynamic oscillatory) strain sweep, frequency sweep, and startup of steady shear. To help relax possible residual stress, the sample was rested for a period of 60 s between two consecutive strains or frequency sweeps and 30 s for the steady-state viscosity (η) measurement. A constant frequency of 1 Hz was utilized in the strain-sweep experiment. The storage (*G*’) and loss (*G*”) modulus obtained in the frequency-sweep experiment utilized a small strain (1%) identified to fall in the linear viscoelastic (LVE) region in a prior strain-sweep experiment. The viscosity measured in the steady-shear experiment was taken from the steady-state value at long times for a given shear rate. The shear rate was increased in a stepwise manner from low to high. To describe the flow behavior of a DFM, the shear stress (σ) versus shear rate (γ) data over a range of shear rates 0.1–100 s^−1^ at 25 °C were fitted to the power-law model to indicate the consistency index (*K*) and flow-behavior index (*n*) as Equation (2); the results were analyzed on a double logarithmic scale, with the values of *K* and *n* determined from the intercept and slope of the plot, respectively [22,34].

*σ* = *K* γ ^*n*^ (2)

### 2.5. Texture Profile Analysis (TPA)

**Figure 1:**
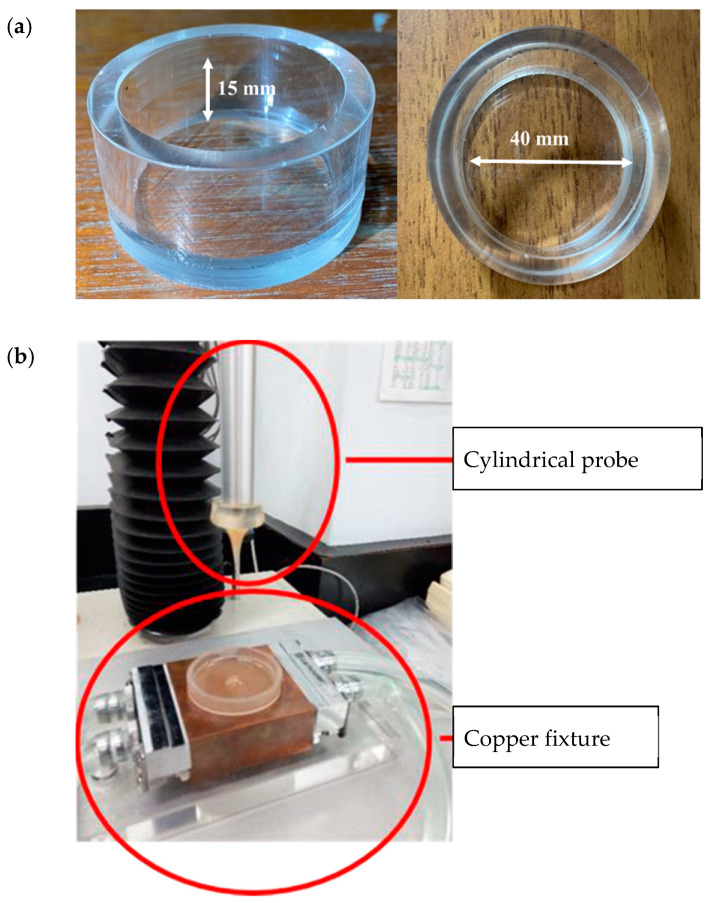
Experimental setup for texture profile analysis (TPA): (**a**) Sample container made of glass with the indicated dimensions. (**b**) Cylindrical probe and the copper fixture holding the glass container that is connected to a circulation system to maintain system temperature at 25 °C throughout the measurement.

The authorized method of TPA issued in 2009 by the Japanese Ministry of Consumers, “Food for patients with swallowing difficulty,” under regulation of “Food for special dietary uses” was adopted for the characterization of model food matrices [18]. A customized glass container (40 mm diameter × 20 mm depth) was filled with the fluid sample, up to the 15 mm level (Figure 1a). The measurement was performed with a cylindrical probe (20 mm diameter) made of acrylic, while the system was maintained at 25 °C using a customized temperature controller (Figure 1b). The texture profile analyzer (TA. XT plus, Stable Micro Systems Co., Ltd., Godalming, Surrey, UK) was programed to measure the hardness, adhesiveness, cohesiveness, and gumminess of each sample; these parameters were calculated using the Exponent Lite software. The copper fixture holding the glass container was positioned directly underneath an acrylic probe with their centers concentrically aligned. The probe was placed 10 mm above the surface of the sample as the starting point and then progressed downward until reaching the 5 mm mark above the bottom of the glass container. The probe was then returned to the starting point, and the same procedure was repeated for a second time. Probing speed was maintained at 10 mm/s throughout the experiment.

### 2.6. Scanning Electron Microscopy (SEM)

Microscopy observations were performed according to a previous study on the structural characterization of hydrogels [35]. The selected DFMs were frozen at XX °C prior to freeze drying. The frozen samples were placed in a benchtop freeze dryer (FD-1C-50, BioCool, Shanghai Bilon Instrument Co. Ltd., Shanghai, China) for lyophilization and thereafter stored in a desiccator with silica gel until microscopy observation. The samples were covered with a platinum layer with thickness of 3–4 nm by a sputter coater. Photomicrographs were obtained using a thermal field emission scanning electron microscope (JSM-7800F, JEOL Ltd., Akishima, Tokyo, Japan) in secondary electron mode at 3 kV accelerating voltage.

### 2.7. Data Manipulation and Statistical Analysis

Each experimental dataset was reported based on triplicates, except for texture profile measurement data and microscopic observations; for texture profile analysis, each experimental dataset was based on nine replications. The regression equations resulting from flow properties and rheological behavior were obtained using SigmaPlot^®^ version 10.0 (SYSTAT software, Inc., San Jose, CA, USA) with percentage R-square indication for the degree of model fitness. The shear rate dependent viscosities with respect to other groups were subjected to paired *t*-test comparisons. Duncan’s multiple range test based on analysis of variance (ANOVA) was utilized to justify the significance of differences among the mean values of the fitted consistency coefficient and flow-behavior index of the corresponding formulated matrices, as well as textural properties with a 95% confidence interval using SPSS (Statistical Package for the Social Science) version 19.0.

## 3. Results and Discussion

### 3.1. Degree of Esterification (DE)

Theoretically, the DE can range from 0% to 100%. Whether a pectin bears a DE above 50% has been used to characterize the high- (or low-) methoxyl pectin [10]. The DE of the AP powder under investigation is 45.2 ± 1.42%. Citrus pectin samples were reported to have DE ranging from 27.9% to 77.8% [22], whereas pectin extracted from sugar beets was reported to range from 61.29 to 70.13% depending on the pH value [18]. Rascón-Chu et al. [36] recently conducted a pectin collecting process by extracting pectin from thinning apple (*Malus demestica* Borkh) with a DE of 41%, which is comparable to our measured value. Pectin substances possess complex *O*-ether or *O*-ester groups, which are contained in the polysaccharide-oriented molecular structure composed of as many as 18 monosaccharides [17]. The AP sample powder recruited in this study appears to be a merely, instead of extremely, low-methoxyl pectin. It could be expected that both sugar associated high-methoxyl effects and divalent calcium ion associated low-methoxyl effects on rheological characterization would be attainable for AP-contained fluid matrices. Moreover, the structural attributes of the AP-contained fluid matrices with the addition of divalent calcium ion can be apparently different from those with the addition of sugar. The DE value could be modified as needed by an alkaline solution, followed by neutralization and a 70% ethanol sedimentation. The DE value could be reduced to as low as 5.6%, an 86% minimizing rate, for pectin made from thinning apple with a 41% DE value [36]. 

### 3.2. Rheological Characterization

#### 3.2.1. Rheological Features of AP at Various Concentrations

**Figure 2:**
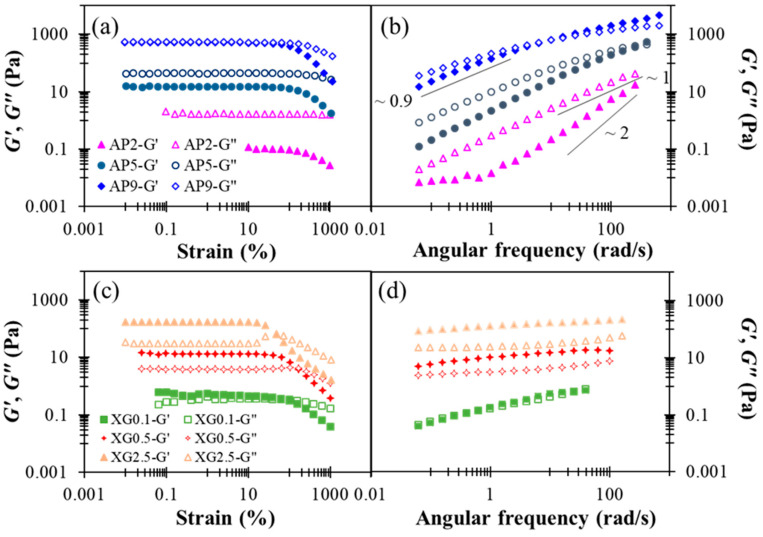
Concentration dependent shear storage modulus (G’) and loss (G”) modulus profiles with respect to percentage strain and angular frequency alterations as for sole thickeners: (**a**) strain sweep of AP, (**b**) frequency sweep of AP, (**c**) strain sweep of XG, (**d**) frequency sweep of XG.

The rheological characterization performed herein may be utilized to simulate the shearing history of model food matrices as well as the corresponding viscoelastic responses during the human swallowing process. In this study, rheological properties of AP-based hydrocolloids are investigated at AP concentrations ranging from 2 to 9% (*w*/*w*). Figure 2a shows the results of a strain sweep. The linear viscoelastic (LVE) region can be seen to hold strains smaller than 100% in all three cases. At low and moderate concentrations (2–5%), the AP-based hydrocolloids exhibited fluid-like behavior with *G*” > *G*’ within the range of strains investigated; there is a pronounced strain-softening for both dynamic moduli at strains exceeding the LVE region. Similar strain-softening behavior was previously reported for a commercial apple purée designated in Level 2 of Japanese dysphagia diet guidelines [18]. As the AP concentration reaches 9%, however, *G*’ and *G*” become almost identical in the LVE region, while *G*’ displays a more prominent strain-softening at still larger strains. Both features mark the onset of active interactions between AP colloids in the semi-diluted or overlapping regime, in agreement with prior observations [37]. According to the results of the strain sweep, a small strain of 0.1 (10%) was chosen for the frequency-sweep experiment to ensure that the microstructure of AP remains intact. For the dilute AP2 sample, Figure 2b shows that the Maxwell-fluid behavior, $G\prime \propto {\;\omega }^{2}$ and $G\prime \prime \propto {\;\omega }^{1}$, is applied, except at low frequencies where a plateau in *G*’ is indicative of the presence of large aggregate species [38]. Zhao et al. [39] recently studied the structural formation of high-methoxyl apple pectin systems without an acidifier; the 1% apple pectin with different DE values and different sucrose levels were subjected to structural investigation. Our frequency sweep results indicate that AP2 hydrocolloid represent a behavior pattern with respect to shear moduli ranging from 0.01–200 (rad/s), which resembles the behavior of 1% high-methoxyl (66.1%) apple pectin contained hydrocolloid with additional 45 and 50% of sucrose undergoing the sweep range between 1–100 rad/s [39]; however, it is generally precautious for the elderly to consume high amounts of sugar for various health concerns. In contrast, the concentrated sample, AP9, exhibits critical-gel behavior [40], ${G\prime \sim G\prime \prime \sim \omega }^{n}\;$(n ≈ 0.9), as often observed for colloidal suspensions just below the percolation or gelation threshold. Likely, these features suggest that the AP hydrocolloids are constituted of colloid-like particles in aqueous solution, and that the AP concentration can offer an excellent opportunity to gain precise control over their viscoelastic properties for various applications with DFMs. 

**Figure 3:**
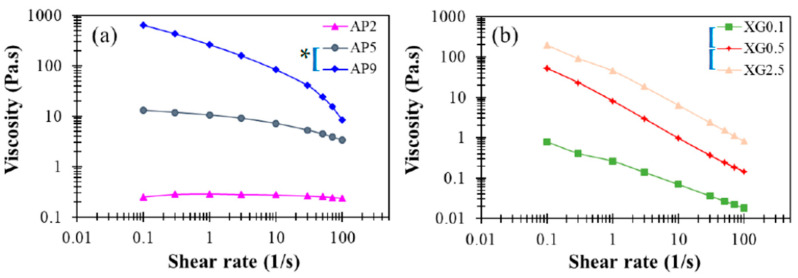
Steady-state viscosity profiles as a function of shear rate for (**a**) AP and (**b**) XG hydrocolloids at various concentrations with viscosity ranging from 0.01 to 1000 Pa.s. The indicated left brackets ([) marked on sample labels are significantly different from each other at the 5% level by a paired *t*-test; the indicated left brackets with an additional star (*[) marked on sample labels are significantly different from each other at the 1% level for a paired *t*-test.

Figure 3a presents the corresponding steady-state viscosity for the range of shear rates 1–100 Hz. While the concentrated sample AP9 exhibits pronounced shear-thinning behavior, the dilute sample AP2 displays Newtonian behavior, as reported in prior work [37]. These features are basically consistent with the dynamic modulus responses dis-cussed in Figure 1b. For a shear rate of 50 s^−1^ that is often employed in the characterization of DFMs, the viscosities of AP hydrocolloids at concentrations of 2%, 5%, and 9% fall around 250, 4440, and 23,980 cp (or mPa∙s), respectively. Such a broad range of viscosity variation supports the notion that AP hydrocolloids may serve as an effective thickener for applications in food industries and nursing facilities to produce desirable DFMs. Statistically, the paired *t*-test indicate significant differences with a 5% or better confidence level for such concentration dependent steady-state viscosities for AP hydrocolloids and specify that only AP2 is possible for DFM formulation because specific viscosity at the shear rate of 50 s^−1^ would be categorized as nectar-like fluid matrices for confinement of the national dysphagia diet standard [41]. Moreover, a recent optimization study [42] reported the steady-state viscosity pattern of leaf pectin with a control sample obtained from a commercial source (FoodPro^®^ CBL manufactured by Dupont company (Shanghai branch, China)), which mainly contained cellulase comparable to citrus pectin with methoxylation degree of 76.68%. Leaf pectin was extracted by different methods—hydrothermal, acidic, and alkaline extractions. Sample concentration was set at 5%. The citrus pectin was found to contain matrices with shear rate dependent viscosity profile ranging from 120–150 mPa∙s, which is lower yet comparable to the AP2-based hydrocolloids we prepared. Zhang et al. [42] also reported the shear moduli with respect to frequency sweep of pectin-based fluids. The G’s of their 5% based sample fluids range from circa 1,000 to 10,000 Pa while G” ranged from circa 200 to 3000 Pa; their reported shear modulus profiles are comparable to our corresponding results of the AP5 hydrocolloid. We also noted that their data was based on the frequency sweeping range of 0.01–100 rad/s, whilst our reported sweeping range was beyond 100 rad/s; the likely critical-gel behavior of 5% based pectin containing hydrocolloid is shown in Figure 2b. 

#### 3.2.2. Time-Concentration Superposition of AP Hydrocolloids

**Figure 4:**
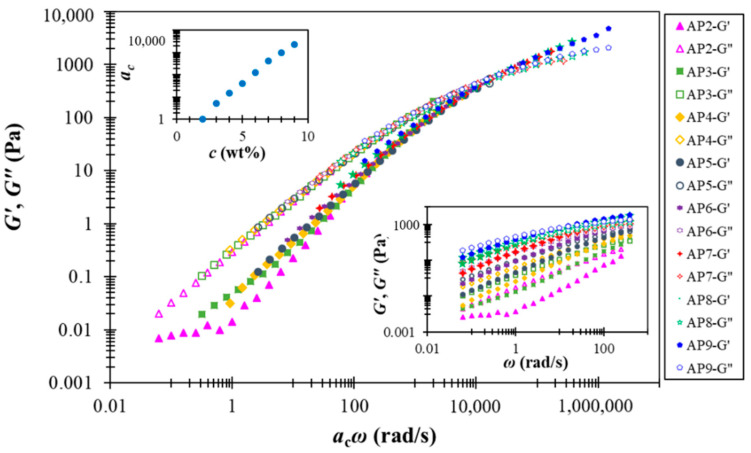
Time-concentration superposition of rescaled dynamic modulus data for AP hydrocolloids in this study. The lower (right) inset presents the original (un-shifted) data, and the upper (left) one shows the shift factor ac as a function of AP concentration.

A notable feature of the AP hydrocolloids in this study is the applicability of time-concentration superposition (TCS), as shown in Figure 4. This feature was first reported for suspensions composed of weakly attractive carbon blacks [43], and a recent interpretation further attributed the underlying physics to the “colloidal meshes” formed under both dilute (dispersed) and concentrated(interconnected) conditions [44]. It thus appears that AP hydrocolloids may have fostered similar (weakly attractive) colloidal species for the range of concentrations investigated. Intriguingly, the resulting master curve covers about eight orders of magnitude in frequency, while the plateau modulus remains basically unchanged. The last feature is in distinct contrast with what has been observed for colloidal suspensions, which typically demonstrate notable concentration-dependent plateau modulus. Although more work is clearly required to resolve the underlying structural features, it seems that AP hydrocolloids share viscoelastic features of both entangled polymer solution and colloidal suspension, making their true attributes highly intriguing. The referred case of weakly attractive carbon black pastes [44] present a somehow likely TCS, however, with the dynamic modulus data over a frequency range of 12 orders of magnitude. The material natures of pectin and carbon blacks are apparently different; yet it is generally believed that soft matter physics principles could alternatively be used to address important problems in the food industry. For example, Wu et al. [45] employed cationic gelatin with anionic pectin at a considerably low concentration (0.01% level) through complex coacervation to mimic swollen starch granules.

#### 3.2.3. Rheological Properties of XG at Various Concentrations

XG is a less-costly thickener when compared to AP and, therefore, has found a wide range of applications. Although XG is the second-most popular dysphagic thickener after modified starches, it is considered more stable because food liquids produced from the latter could be vulnerable to hydrolysis by the salivary amylase, in addition to the notable fluctuation in viscoelastic properties during storage [18]. Herein, the XG hydrocolloids are prepared at concentrations ranging from 0.5 to% to characterize their rheological behavior. 

Figure 2c shows the dynamic modulus responses during strain sweep. All the XG hydrocolloids investigated exhibit gel-like attributes (*G*’ > *G*”) in the LVE region, and the most concentrated sample, XG2.5, additionally produces a slight overshoot in *G*” at still larger strains; this has been classified as a “weak strain overshoot” as per a prior study [46]. These rheological features of XG hydrocolloids are basically in agreement with a previous report [47]. The results of the frequency sweep shown in Figure 2d further indicate that, with increased XG concentration, the hydrocolloid may be converted from a critical gel (*G*’ ≈ *G*”) to a strong gel (*G*’ >> *G*”), exhibiting little dependence on angular frequency. It was recently documented that for a 0.694%XG contained matrix of distilled water base that underwent a 6 min thermal process at 80 °C; the *G*’ and *G*” remained consistent at the approximate range of 10 Pa [23], which is comparable to the present work of XG 0.5. They also present shear moduli of 4.17% hydroxyl distarch phosphate (a resistance starch approved for use in the European Union with the listed number of E1442) in a distilled water based hydrocolloid that underwent a 3-min thermal process at 80 °C; both moduli are slightly higher than those of 0.694% XG contained matrix with a G’ range less than 200 Pa. It can be deduced that by adequately administrating the recommended amount of water without calorie supplementation concerns, sole xanthan gum can be comparable to certain starch base matrices. 

The corresponding steady-state viscosity shown in Figure 3b demonstrates profound shear-thinning behavior, which is often deemed favorable for food processing. When compared to AP hydrocolloids, the XG counterparts clearly provide proper elasticity to liquid food, even at rather low dosages (e.g., *c* = 0.05 %). As such, XG may, in practice, serve as an efficacious rheological modifier of AP-based DFMs, while reducing the cost of production by reducing the amount of AP used. Overall, the contrasting rheological features noticed between AP and XG hydrocolloids suggest that they may play complementary roles in producing food products for various applications with DFMs. The apparent viscosity can be measured by a rotary viscometer with recommend shear rate of 50 s^−1^; it was previously reported that apparent viscosity of a 0.694% XG contained matrix is 307.1 ± 936 mPa∙s [23], which is comparable to our XG0.5 data of 238.9 ± 5.61 mPa∙s, as well as our AP2 data of 238.2 ± 4.82 mPa∙s. 

#### 3.2.4. Effects of Basic Formulation Ingredients (BFIs) on AP and XG Hydrocolloids

Sugar and some basic formulation ingredients (BFIs) containing cations, such as Ca^2+^ and Na^+^, may add modifications to the rheological properties of AP and XG hydrocolloids, considering especially their ubiquitous presence in liquid foods. Thus, we employ the benchmark AP2 and XG0.5 hydrocolloids containing granulated sugar (with sucrose as the positive control), table salt (with sodium chloride as the positive control), or calcium chloride to shed light on their effects on the rheological properties of AP and XG hydrocolloids.

**Figure 5:**
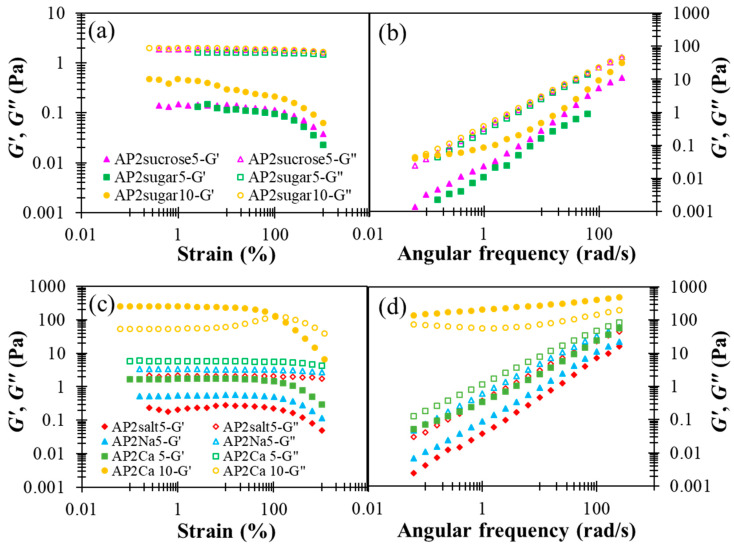
The effect of designated basic food ingredients (sugars and cations) incorporated AP2 based formulation on shear storage modulus (G’) and shear loss (G”) modulus profiles with respect to percentage strain and angular frequency alterations: (**a**) strain sweep of sugar or sucrose incorporation, (**b**) frequency sweep of sugar or sucrose incorporation, (**c**) strain sweep of monovalent sodium (table salt or chemical grade sodium chloride) and divalent calcium, (**d**) frequency sweep of monovalent sodium and divalent calcium; BFIs concentrations are either 5 or 10%.

The results presented in Figure 5, in general, reveal the notable impact of BFIs on the rheological properties of AP2 hydrocolloids, when compared with the results for pure AP2 in Figure 2. First, pure sucrose and sodium chloride possess minor yet appreciable effects compared to their formulation ingredients, i.e., granulated sugar and table salt, especially on the elastic modulus *G*’. While the addition of sugar basically does not alter the fluidity *G*”, as shown in Figure 5a,b, both *G*’ and *G*” can be seen to be modified appreciably with the addition of salt, as shown in Figure 5c,d. The reasons for this disparity remain unclear. Figure 5a,b also reveal that doubling the sugar concentration from 5 to 10% leads to a substantial promotion in *G*’, and this phenomenon might be attributed to the fact that sugar helps foster larger and more compact AP clusters through hydrolyzed bonding. The effects of salt or cation are discussed in more detail below. 

**Figure 6:**
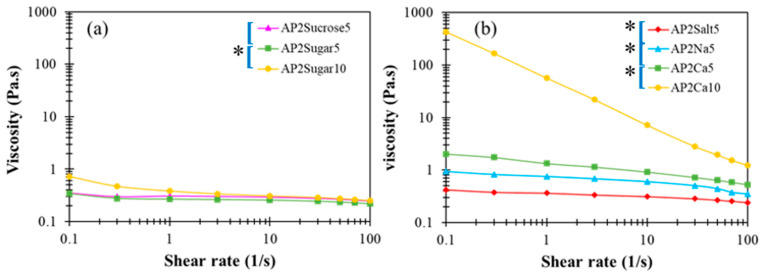
Steady-state viscosity profiles as a function of shear rate for AP-based matrices with (**a**) sugar incorporation and, (**b**) cations at either 5 or 10 % concentrations; the indicated left brackets ([) marked on sample labels are significantly different from each other at the 5% level by a paired *t*-test; the indicated left brackets with an additional star (*[) marked on sample labels are significantly different from each other at the 1% level by a paired *t*-test.

In the literature, divalent calcium ions have been proposed to interact with carboxyl groups to form two adjunctive bridges between the major backbones of pectin molecules, referred to as the “egg box” structure [48]. The detailed interactions between AP and Ca^2+^ as well as the resulting multi-scale structural features remain unclear at present. In Figure 5c,d, a critical amount of divalent calcium associated with AP2Ca10 can be seen to fundamentally alter the attribute of a dilute AP hydrocolloid from fluid-like to gel-like, when there are more than order-of-magnitude changes in both dynamic moduli. A similar observation applies to the steady-state viscosity shown in Figure 6, revealing that only the APCa10 exhibits simultaneously high viscosity and pronounced shear-thinning behavior. Although the rest of AP2 hydrocolloids show less pronounced changes, they clearly offer a variety of choices for fine-tuning the viscosity of the AP-based DFMs. It is worth noting that the rheological properties of XG hydrocolloids are basically unaffected by the addition of various types of BFIs (results not shown here). This major disparity between AP and XG, again, points to the open opportunity of their individual utilization and combination to meet various requirements in future applications with DFMs.

#### 3.2.5. Rheological Behavior of DFMs with a AP-XG Composite (Blend) Thickener

**Figure 7:**
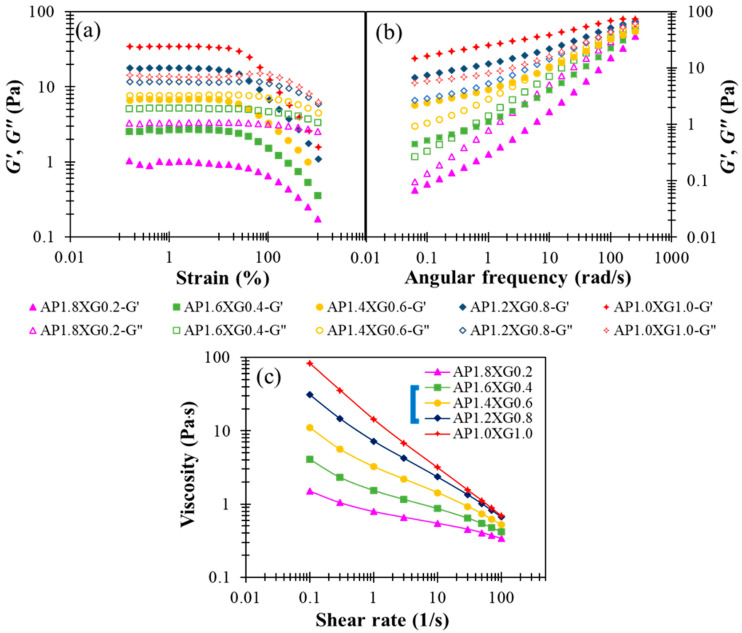
The modification effect of XG with regards to shear storage modulus (G’) and shear loss (G”) modulus profiles on AP-based DFMs under the designated AP-XC composite constraint of 2%: (**a**) strain sweep of AP-XG composite contained DFMs, (**b**) frequency sweep of AP-XG composite contained DFMs, and (**c**) the steady-state viscosity as a function of shear rate for various AP-XG composite contained DFMs.

Designing liquid food formulations for dysphagic care often requires the elastic and viscous attributes associated with a specific composite ratio to be adequately compromised, because too high an elastic consistency could obstruct the mixing of food bolus with saliva [49]. Thus, we consider various combinations of AP-XG for their complementary viscoelastic features explored above, while fixing the total concentration at 2%. The following formulas are investigated: AP1.8XG0.2, AP1.6XG0.4, AP1.4XG0.6, AP1.2XG0.8, and AP1.0XG1.0. It can be seen from Figure 7a,b that as the dosage of XG0.6 is reached, the hydrocolloid turns from liquid-like to gel-like. Note, however, that the weak strain overshoot in *G*” previously observed with XG2.5 (Figure 2c) and AP2Ca10 (Figure 5c) becomes barely appreciable, suggesting that the elastic attribute of the AP-XG hydrocolloids changes from that of a weak gel for typical colloidal suspensions to that of semi-dilute polymer solutions. The actual mechanism and underlying microstructure leading to this alteration remain to be explored. The steady-state viscosity shown in Figure 7c exhibits shear-thinning behavior for all AP-XG combinations, even at a low dosage of XG0.2 (recall that Figure 3a reveals Newtonian behavior for AP2). The steady-state viscosity measured at 50 s^−1^ varies from 410 cp (for AP1.8XG0.2) to 1120 cp (for AP1.0XG1.0), all conforming to a honey-like consistency [3]. This feature along with the versatile viscoelastic properties of AP-XG hydrocolloids should motivate their future applications with DFMs. Ortega et al. [50] examined a commercial composite thickener, which is claimed to be composed of starch and xanthan gum as the formula base (ratio not revealed); four different phenotypes of patients with dysphagia were tested: elderly without a chronic disease diagnosis, with head/neck cancer, with Parkinson’s disease, and with chronic poststroke. Upon preparing the sample matrices using the referred starch-gum based composite, the matrices generally presented shear-thinning behavior when submitted to shear, which resembles our prepared samples. Their prepared shear viscosity 25–100 mPa∙s scores a safe swallow ratio ranging between 74.19–96.67% by the noncancer groups (58.6% by the neck/head cancer group). Impressively, none of the prepared matrices (shear viscosity ranging from 25 to 2000 mPa∙s) registers α-amylase degradation after 30 s oral incubation. Deductively, certain types of resistant starch or other likewise cross-linked carbohydrates could possibly be recruited into the referred composite formula. Gallegos et al. [51] reported that in less than 30 s in the oral phase of swallowing, the viscosity of starch-based thickeners can be drastically reduced due to the enzymatic degradation of α-amylase contained in saliva. Therefore, our AP-XG composite would be of great potential when α-amylase resistance is a concern. 

#### 3.2.6. Flow Behavior Characterization

The power-law model is commonly used to classify the flow behavior of fluid-oriented food matrices. The parameters in the power-law model are closely related to the human swallowing process, wherein *K* is related to the propagation velocity of a food bolus and *n* reflects the smoothness of the food bolus contained in the oral cavity when conveying through the pharyngeal passage [18,52,53]. The regression indices of *R*^2^ (93.32–99.99%) in Table 2 indicate generally excellent fit of the power-law model. In fact, a relatively large value of *K* for XG-based hydrocolloids is known to enhance the sliminess and compactness of food bolus, which is unfavorable for dysphagic individuals. Thus, as a good DFM candidate, it is often necessary to reduce the concentration of XG or to blend it with other low-*K* hydrocolloids [52,54]. Yang and Lin [23] recently incorporated 0.467–0.694% of XG into different aquatic bases of distilled water, sport drink, and orange juice; the *K* and *n* values from regression model range between 6.726–7.554 and 0.143–0.177, respectively, and agree with the present study comparing to XG0.5; they also reported the fitness regarding the regression models of Herschel-Bulkley, power law, and Casson. Their modeling results indicate that the terms of yield index are present in negative values for Herschel-Bulkley regression and, are not applicable to the XG-based matrices in our experiment range. In addition, Yoon and Yoo [22] documented that the power law and Casson models can both fairly present the flow behaviors of a composite thickener (combination of XG, guar gum, and dextrin without revealing the composite ratio) for concentrations of at least 1% and give a considerable low flow-behavior index of 0.24 (*n*). Therefore, we consider that the sole power law modeling can adequately present behaviors for XG-contained matrices up to the designated level with additional *n* values, which are not presented as the Casson model is used.

In Table 2, the flow-behavior index (*n*) for the AP and XG hydrocolloids without inclusion of BFIs can be seen to vary in a wide range—from 0.1233 to 0.9689—and, therefore, may well be classified as non-Newtonian fluids (*n* < 1) [22]. It is reported that XG-based matrices with approximate 0.5% (*w*/*w*) concentration level would have an *n* range of 0.14–0.18, indicating a preferable mouthfeel [23]. Interestingly, AP2Ca10 also presents a comparably low *n* of 0.124 ± 0.0089; compared to the AP2 and AP2Ca5 contained matrices, an implication almost convincing enough is that divalent calcium helps improve mouthfeel in terms of the flow-behavior index. The values of *n* can also be seen to decrease with the increased concentration of AP or XG. In contrast, the consistency coefficient (*K*) for both AP and XG hydrocolloids apparently enlarges with increased concentration. Previously, it was reported that as the solid content in goat milk increases, the corresponding value of *n* decreases [55]. For purée products made of fruits and vegetables, the solid contents were noticed to have a strong effect on the values of both *K* and *n*, and a higher solid content generally yields a larger *K* and smaller *n* [56,57]. Divalent metal salts are particularly considered to associate themselves with pectin molecules in purée and result in weak gels [58]. Clearly, the AP2Ca10 in this study exhibits the smallest *n* as well as the largest *K* (except for AP9) among the AP-based hydrocolloids investigated. We note in passing that a larger *K* usually implies the requirement of a longer time or greater force to modify food consistency.

For AP-XG composite (blend) hydrocolloids, *K* is found to decrease while *n* increases with increasing AP content; a similar trend was noticed with tapioca starch when employed as a dysphagia-friendly formulation [23,56]. Thus, AP may serve as an alternative thickener to replace common starches. Still others have reported that the flow behavior of XG can be modified by increasing the polysaccharide content to enhance the association between the biopolymers in a hydrocolloid system [59]. It is believed that AP-XG blend thickeners alone or with the addition of some BFIs may help achieve functions associated with starch-based DFMs, while providing other health benefits of AP (e.g., in preventing diabetes and regulating cholesterol level; AP has been classified as a source dietary fiber [18]). 

### 3.3. Texture Profile Analysis (TPA)

In addition to the rheological characterization, TPA provides other important measurements of a dysphagia-friendly matrix (DFM). In this study, TPA is conducted by applying a vertical pressing force to mimic the human oral chewing process, and the setup and protocols follow those recommended by the Japanese Ministry of Health and Welfare in Japanese [60], first revealed by Funami [18] and, thereafter reviewed and discussed by Matsuo and Fujishima [61]. The characterization utilizes the standard parameters of hardness, adhesiveness, and cohesiveness. Hardness represents the maximum force required to crash or break a food bolus and is related to the maximum velocity of swallowing. Note, however, that according to the Japanese Ministry of Health and Welfare, hardness is measured in terms of N/m^2^ (pressure) rather than newton (force). The glass container (40 mm in diameter) employed in the characterization bears the dimensions of the human oral cavity. Adhesiveness is defined by the degree of sticking or adhering between the food bolus and the upper swallowing system, which includes the oral cavity and pharyngeal mucosa. Cohesiveness is a measure of interior binding strength, which constructs bolus tightness to prevent its collapse during swallowing. Gumminess, which is related to the effort required for preparation of fluid-oriented bolus by the oral cavity, can then be evaluated by multiplying the measured hardness by the corresponding cohesiveness [62]. Several studies have included gumminess as a major textural index of certain food samples. One set of mixed gel was prepared by Huang et al. [63] using sticky rice with a composite thickener and CaCl_2_ for the preparation of an aquatic phase, which is subjected to textural studies, including gumminess. Momosaki et al. [64] also reported the statistically positive correlation between gumminess of semi-solid foods and post-stroke dysphagic individuals. Carrot purees were subject to textual modification with thickeners, including pectin xanthan, thereafter, and were proven to be correlated well with certain sensory attributes [65]. 

**Figure 8:**
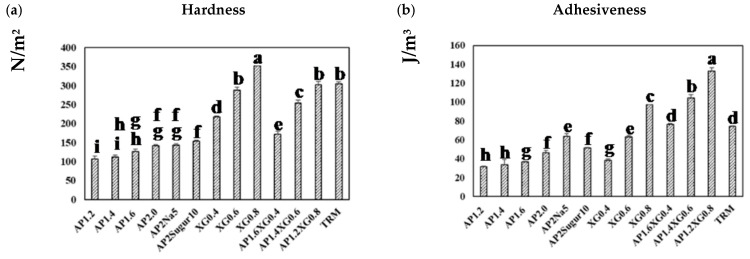

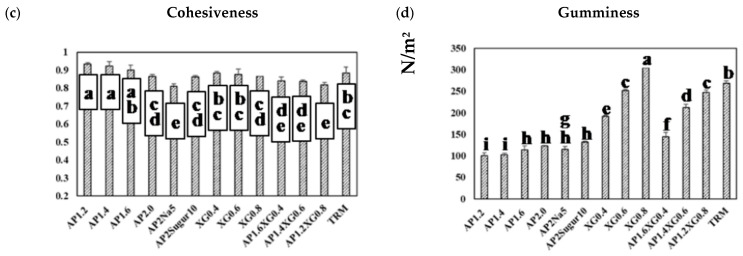
Texture profile analysis (TPA) on a series of model hydrocolloids: (**a**) hardness, (**b**) adhesiveness, (**c**) cohesiveness, and (**d**) gumminess. Error bars represent the standard deviations based on nine replicates. The lowercase letters indicate significant difference (*p* < 0.05) of the referred textural properties; the x-legends represent the data of sole AP matrices, sodium or sugar contained AP matrices and composite AP-XG matrices, and the matrix prepared by a commercialized product, namely Neo-high Toromeal III^®^, as control.

Figure 8a presents the hardness of a representative series of hydrocolloids. The AP-based (*c* = 1.2–2.0%) and XG-based (*c* = 0.4–0.8%) samples both reveal a concentration-dependent hardness, and the latter can be seen to contribute notably to the hardness, even at low concentrations. With the total concentration fixed at 2%, the hardness of AP-XG hydrocolloids increases substantially with increased dosage of XG. Especially, the hardness of the AP1.2XG0.8 successfully reaches the threshold value of 300 N/m^2^ [3] for dysphagia-friendly food bolus according to the criteria recommended by Japanese authority CRJA [18]. The measured hardness values of hydrocolloids prepared from the control sample, Neo-high Toromeal III^®^, and AP1.2XG0.8 based hydrocolloids, are 304.66 ± 5.773 and 302.00 ± 9.849 N/m^2^, respectively; both these measured values are not significantly different (*p* > 0.05) from the sole XG0.6 thickened hydrocolloid, even through its measured hardness is slightly below the threshold value of 288.33 ± 7.506 N/m^2^. It can also be seen that while the addition of table salt (AP2Na5) has little change in hardness, the promotion becomes noticeable with the addition of sugar (AP2Sugar10) for AP-based hydrocolloids. 

The corresponding results on adhesiveness are presented in Figure 8b. A pronounced increase in adhesiveness for AP-based hydrocolloids can be observed as the AP concentration is raised above 1.6%. Both table salt and sugar help further promote the adhesiveness of the AP-based hydrocolloids, with the former showing a more prominent effect. The adhesiveness of the XG-based samples exhibits a nearly linear growth with the XG concentration, and the combined AP-XG formulas generally show high adhesiveness as well. All formulations under investigation comply with the CRJA (<1500 J/m^3^) [18]. In contrast, the cohesiveness in all cases (Figure 8c) falls in a narrow range of 0.8~0.9 and complies with the CRJA (0.2–0.9) [18]. Accordingly, the gumminess (Figure 8d) of the investigated samples can be expected to be governed by their hardness.

Because TRM is a commercialized thickener widely prescribed to dysphagia individuals, its results are included in Figure 8 for comparison. It should be evident that the formulations based on AP alone are nowhere close to TRM (1.5%) in the TPA characterization. On the other hand, AP1.2XG0.8 and TRM appear to possess similar hardness, cohesiveness, and gumminess, although the former produces a considerably greater adhesiveness; both formulations comply with CRJA [18]. 

Park et al. [66] indicated that the attempts to increase viscosity can reduce the risk of aspiration in the airway; however, food with excessive thickness requires more force and effort for a smooth swallowing process; thus, they investigated the texture of nine swallow-friendly foods that do not require mastication for texture profile analysis. The geometry and diameter of the TPA probe as well as the test protocol we used resemble that of a previous study [66]. As we have taken probe dimensions into account, the dimensions involving TPA parameters of hardness and adhesiveness are comparable by trends but different in units between the present study and the mentioned study; however, the dimensionless TPA parameter of cohesiveness is directly comparable between the two studies. The cohesiveness of mayonnaise and peanut butter is reported to be 0.87 ± 0.03 and 0.8 ± 0.04, respectively; these values are comparable to our most employed samples, while other test samples, including whipped cream, soft tofu, mango pudding, boiled mashed starchy vegetable (pumpkin, potato, and sweet potato), and red bean paste possess considerably lower cohesiveness, compared to our AP-based matrices. By converting the present hardness into area-dependent data, 350 N/m^2^ is approximately 0.44 N; and the values apparently fall between the measured values of 0.26 ± 0.03 and 0.63 ± 0.02 N for whipped cream and mayonnaise, respectively. It should be noted that the hardness unit of N/m^2^ is consistently used for categorization of dysphagia-friendly foods CRJA [18]. 

### 3.4. Scanning Electron Microscopic (SEM) Analysis

**Figure 9:**
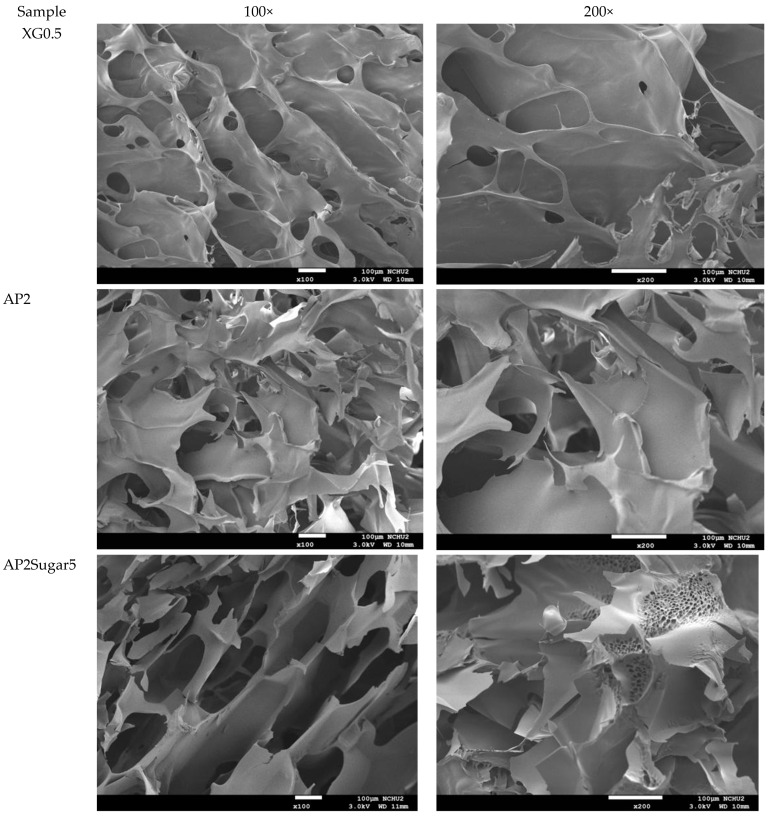

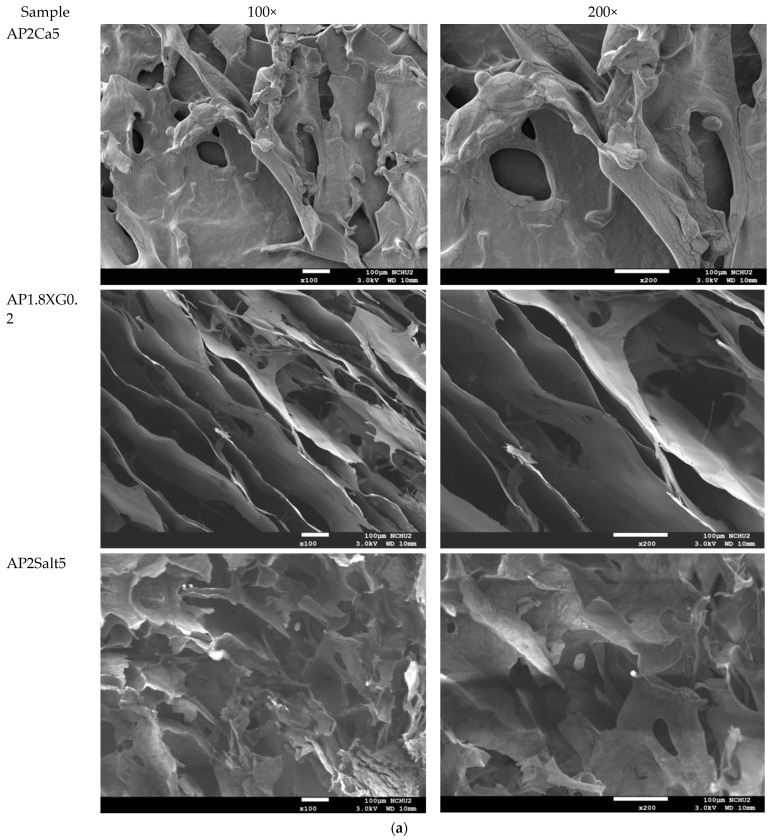

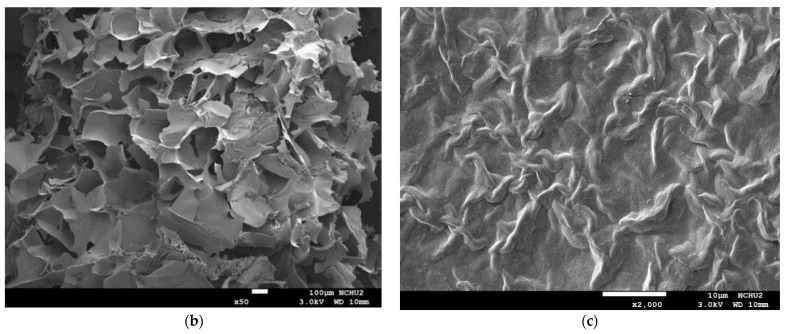
Scanning electron microscopic images of lyophilization sample matrices: (**a**) Sample with magnified levels of 100× and 200× for the samples of XG0.5, AP2, AP2Sugar5, AP2Ca5, AP1.8XG0.2, and AP2Salt5, respectively; (**b**) The 50× image of AP2Suagr5 evidencing a hive-like structure corresponding to 200× level in (**a**); (**c**) The 2000× image of AP2Salt5 evidencing possible isolated salting particles.

The analysis of the internal microstructure by scanning microscopy was performed for select DFMS—the ones containing a single thickener (XG0.5, or AP2 along), AP2 with BFIs (AP2Suagr5, AP2Ca5, and AP2Salt2), and the composite thickener formulation of AP1.8XG0.2. The photomicrographs are shown in Figure 9. Generally, there is evidence of a porous structure for all the lyophilized DFMs. Through the images, we observed an obvious three-dimensional microstructure interconnected by virtue of the expedite lyophilization process with the pores having ice crystal formation. In the light of structural smoothness, it is apparent that both the calcium ion sample (AP2Ca5) and the composite thickening formulation (AP1.8XG0.2) with XG incorporated into AP possessed smoother surfaces than the formula with singular thickener samples. In addition to the smoothness, in the 200× magnified microstructure image of the AP2Ca5 contained sample, some clear vein-like streams appear on the network surface; meanwhile, the calcium could possibly be incorporated into the dispersed phase to result in a much thicker layer compared to other investigated samples; otherwise, the same ionic crystal can be observed as shown in 2000× (Figure 9c) magnified microstructural image of a salt-contained sample. By incorporating XG into a hydrogel system, there would expectedly be evidence of cross-linking between molecules, resulting in somehow more compact gaps [67]. The structural modification effect of XG has been reported previously for a millet-based matrix with the incorporation of whey protein and XG as thickening agents [68]; de Alcântara et al. [35] also indicated such a modification effect of XG as electrostatic hydrogels by acidified gelatin and carrageenan. However, there has been rare documentation reporting the XG modification effect on AP matrices; it would be interesting to elaborate on this in this detail. The microstructures of AP-based DFMs with the inclusion of sugar and calcium are rather distinctive. By comparing the differences between the 200× magnified images of AP2Sugar5 and AP2Ca5, we observed for the former scattered sub-sections with even, small porous clusters (Figure 9b) resembling a beehive, in contrast to the thickened layers of the AP2Ca5 sample. The hive-like microscopic image of AP2Suagr5 could somehow be related to structure formation mechanisms in pectin gelation, i.e., (I) hydrogen bonds between undissociated carboxyl groups, and (II) hydrophobic interactions [17,69]. The thick, or literally plump microscopic image of AP2Ca5 sample associations constitute junction zones, popularly known as the “egg box model” [48], responsible for gel formation. Pairs of helical chains are generally considered to be packed with calcium ions located between supplementing oxygen atoms from the guluronate chains involves in the coordination of the calcium ions [48,70]. Braccini and Pérez further revisited the egg-box model on a molecular basis by corroborating two stages in the calcium-involved gelation mechanism, i.e., stage (I): the strongly linked dimer associations, the stage (II): weak inter-dimer associations dominated by electrostatic interactions [70]. The vein-like microscopic image of the AP2Ca5 sample is possibly an indication of the impaired weak associations during the lyophilization process. Einhorn-Stoll [71] recently showed examples of typical real particle SEM images from different pectin preparations, including commercial high-methoxyl pectin along with three low-methoxyl pectins via alkaline, acidic, and enzymatic demethoxylation with a 500× magnified magnitude. The SEM images indicate that the commercial high-methoxyl, alkaline demethoxylated low methoxyl, and acidic demethoxylated low methoxyl pectins are more or less rounded; however, they differ in their surface images. These images can somehow be corelated to the image of our calcium-associated sample. 

The selected samples for SEM observation are restricted by IDDSI (international dysphagia diet standardization initiative) preparation guidelines [18]. The SEM images also reasonably support the rheological measurements. It should be noted that we employed a comparatively low gap measuring geometry (55 mm) for the rheological measurement; there is a possibility that the properties of the thickeners were measured rather than the properties of the continuous phase. However, the 100× SEM image proved that network layers of the continuous phase (hollow parts) are in the order of 100 µm, which can be measured for the employed gap size. Furthermore, the evenly distributed network structure of the selected AP contained samples also demonstrated that the observed DFMs are likely to be fully miscible by comparing the previously reported SEM images of assorted pectin powder particles [71]. 

### 3.5. Justification of Application Limitations and Future Works

The rheological modification effects of XG on starch-based matrices by recruiting natural or modified starch sources have previously been reported [22,23,24]. There could be a possible alternative ingredient choice for a pectin or pectin-based composite thickening formulation with xanthan modification due to the void of retrogradation after gelatinization; therefore, a parameter such as sedimentation would be of the least concern. Additionally, pectin and pectin-based composite materials have recently been considered to have promising biomedical/pharmaceutical applications (prebiotic effects, glucose regulation, insulin delivery, etc.) beyond food texture modification attributes [26]. Our developed thickener choice could potentially serve as a dysphagia-friendly formulation with respect to international guidelines: AP2, AP2Sugar5, AP2Ca5, and AP2Salt5 as level 2; AP1.8XG0.2 as level 3; and XG0.5 as level 4. It is important, however, to conduct video-fluoroscopy to corelate the viscoelastic properties of dysphagia-friendly matrices to dysphagia severities, prior to the product being commercially available [72]. Although this commercial product had been subjected to texture profile analysis, such videoscope technology will be introduced in our future study for safe validation in human consumption. Our proposed AP-based composite could be deemed economically and environmentally sustainable while focusing on the major source being apple pomace [16]. However, feasibility challenges should be studied to understand the efficiency of the AP extraction approach; and optimization studies should be conducted, especially on operational cost issues. Other research challenges would be the optimization of the formulation with a more complex module of AP-XG composite blending sugar and/or cations that are to be fully miscible in different aquatic continuous phases, for example, the acidified ones. Future research will possibly focus on the evaluation of AP composites for the controlled delivery of drugs—which has recently found increased interest [26]—as individuals with dysphagia find it impossible to take orally administrated drugs and thin liquids.

## 4. Conclusions

AP hydrocolloids are shown to exhibit fluid-like behavior at low and moderate concentrations and turn into weak gels (or critical gels) above 9%. The resulting dynamic modulus data (2–9%) exhibit excellent time-concentration superposition over eight orders of magnitude in frequency, a phenomenon often associated with weakly attractive colloidal systems but previously unreported for AP systems. The addition of common BFIs can notably promote the elasticity of the AP hydrocolloids without majorly altering the viscous attribute. The most prominent effect was identified with divalent calcium with a threshold concentration of 10%. XG hydrocolloids were seen to serve as an effective and inexpensive modifier for AP-based hydrocolloids, as they can substantially promote the elastic modulus, even at rather low dosages (<1%) and remain largely unaffected by the presence of common BFIs. The AP-XG hydrocolloids at a constrained total concentration of 2% exhibit the capability for further modification with respect to the viscoelastic attributes that were previously unobserved with non-composite (pure) AP and XG hydrocolloids. The composite AP1.2XG0.8 presents a measured hardness almost identical to that of the commercialized product, namely, Neo-high Toromeal III^®^, with similar cohesiveness and gumminess; the discrepancy of the adhesion could possibly be modified by XG or BFIs. AP2, AP2Sugar5, AP2Ca5, AP2Salt5, AP1.8XG0.2, and XG0.5 are dysphagia-friendly formula bases with respect to viscosity consideration. About 5 or 10% divalent calcium in an AP2-based matrix offers better mouthfeel.

## Figures and Tables

**Table 1:** Summary of the thickeners, sugars, and cations in terms of concentrations and abbreviation for dysphagia-friendly potential evaluation.

Thickener	(%, *w*/*w*)	BFI	(%, *w*/*w*)	Abbreviation
Apple pectin (AP)	2–9	-	-	AP2-AP9
	2	Table salt	5/10	AP2Salt5
		Sugar granule		AP2Sugar5
		NaCl		AP2Na5
		Sucrose		AP2Sucrose5
		CaCl_2_		AP2Ca5/10
Xanthan gum (XG)	0.05–2.5	-	-	XG0.5-XG2.5
	0.5	NaCl	5/10	XG0.5Na5
		Sucrose		XG0.5Sucrose5
		CaCl_2_		XG0.5Ca5/10
Combined AP-XG *	AP1-1.8 + XG1-0.2	-	-	AP1XG1 through AP1.8XG0.2

* The total concentration of AP-XG composite formula is constrained at 2% (*w*/*w*).

**Table 2:** Regression models of power law for thickened fluid matrices formulated with the sole thickener of AP or XG, AP incorporating sugar or cations, and a AP-XG composite *.

Sample	*K* (Pa·s*^n^*)	*n*	*R*^2^ (%)
AP2	0.281 ± 0.0029 ^D,h^	0.974 ± 0.0040 ^A,abc^	99.96
AP5	9.718 ± 0.0712 ^C,e^	0.802 ± 0.0037 ^AB,bcd^	99.67
AP9	233.574 ± 4.2723 ^A,a^	0.414 ± 0.0107^AB,cde^	92.54
XG0.1	0.253 ± 0.0051 ^D,h^	0.432 ± 0.0012^AB,cde^	99.93
XG0.5	7.517 ± 0.0890 ^C,f^	0.128 ± 0.014 ^B,f^	96.78
XG2.5	37.258 ± 0.154 ^B,c^	0.191 ± 0.0343 ^B,ef^	93.32
AP2Sugar5	0.270 ± 0.0016 ^C,h^	0.958 ± 0.0035 ^A,a^	99.99
AP2Sugar10	0.532 ± 0.0037 ^A,h^	0.817 ± 0.0023 ^B,ab^	99.13
AP2Sucrose5	0.316 ± 0.0009 ^B,h^	0.952 ± 0.0042 ^A,a^	99.98
AP2Salt5	0.348 ± 0.0015 ^C,h^	0.934 ± 0.0019 ^A,a^	99.96
AP2Na5	0.727 ± 0.0023 ^C,h^	0.862 ± 0.0024 ^B,a^	99.84
AP2Ca5	1.379 ± 0.0086 ^B,h^	0.798 ± 0.048 ^C,ab^	99.97
AP2Ca10	64.401 ± 0.5832 ^A,b^	0.124 ± 0.0089 ^D,f^	98.11
AP1XG1	16.034 ± 0.0472 ^A,d^	0.318 ± 0.0102 ^E,def^	99.56
AP1.2XG0.8	8.282 ± 0.0374 ^B,ef^	0.452 ± 0.0086 ^D,cde^	99.59
AP1.4XG0.6	3.791 ± 0.0256 ^C,g^	0.569 ± 0.0094 ^C,bcd^	99.63
AP1.6XG0.4	1.787 ± 0.0147 ^D,h^	0.688 ± 0.0024 ^B,abc^	99.74
AP1.8XG0.2	0.903 ± 0.0112 ^E,h^	0.791 ± 0.0086 ^A,ab^	99.90

* Shear rates for all regression models range between 0.1–100 Hz. Values are mean ± standard deviation; n = 3. *K* indicates the consistency coefficient and *n* indicates the flow-behavior index of the corresponding formulated matrices. The uppercase letters indicate significant differences (*p* < 0.05) within the sub-group (differentiated by grey rows). The lowercase letters indicate significant differences (*p* < 0.05) in the whole group.

## Data Availability

Data are contained within the article or supplemented upon request from the section editors.
